# Congenital infection with atypical porcine pestivirus (APPV) is associated with disease and viral persistence

**DOI:** 10.1186/s13567-016-0406-1

**Published:** 2017-01-06

**Authors:** Lukas Schwarz, Christiane Riedel, Sandra Högler, Leonie J. Sinn, Thomas Voglmayr, Bettina Wöchtl, Nora Dinhopl, Barbara Rebel-Bauder, Herbert Weissenböck, Andrea Ladinig, Till Rümenapf, Benjamin Lamp

**Affiliations:** 1 Department for Farm Animals and Veterinary Public Health, University Clinic for Swine, University of Veterinary Medicine Vienna, Veterinaerplatz 1, 1210 Vienna, Austria; 2 Department of Pathobiology, Institute of Virology, University of Veterinary Medicine Vienna, Veterinaerplatz 1, 1210 Vienna, Austria; 3 Department of Pathobiology, Institute of Pathology and Forensic Veterinary Medicine, University of Veterinary Medicine Vienna, Veterinaerplatz 1, 1210 Vienna, Austria; 4 Traunkreis Vet Clinic, Großendorf 3, 4551 Ried im Traunkreis, Austria

## Abstract

In 2013, several Austrian piglet-producing farms recorded outbreaks of action-related repetitive myoclonia in newborn piglets (“shaking piglets”). Malnutrition was seen in numerous piglets as a complication of this tremor syndrome. Overall piglet mortality was increased and the number of weaned piglets per sow decreased by more than 10% due to this outbreak. Histological examination of the CNS of affected piglets revealed moderate hypomyelination of the white substance in cerebellum and spinal cord. We detected a recently discovered pestivirus, termed atypical porcine pestivirus (APPV) in all these cases by RT-PCR. A genomic sequence and seven partial sequences were determined and revealed a 90% identity to the US APPV sequences and 92% identity to German sequences. In confirmation with previous reports, APPV genomes were identified in different body fluids and tissues including the CNS of diseased piglets. APPV could be isolated from a “shaking piglet”, which was incapable of consuming colostrum, and passaged on different porcine cells at very low titers. To assess the antibody response a blocking ELISA was developed targeting NS3. APPV specific antibodies were identified in sows and in PCR positive piglets affected by congenital tremor (CT). APPV genomes were detected continuously in piglets that gradually recovered from CT, while the antibody titers decreased over a 12-week interval, pointing towards maternally transmitted antibodies. High viral loads were detectable by qRT-PCR in saliva and semen of infected young adults indicating a persistent infection.

**Electronic supplementary material:**

The online version of this article (doi:10.1186/s13567-016-0406-1) contains supplementary material, which is available to authorized users.

## Introduction

Congenital tremor (CT) of piglets is a common phenomenon characterized by a generalized shaking involving the whole musculoskeletal apparatus. CT is generally classified in two types of disease. While histopathological lesions are missing in type B, the type A is associated with variable hypomyelination of brain and spinal cord. These histological lesions are found as inherited genetic defects in male Landrace pigs in type A-III [1] and in Saddleback pigs in type A-IV [2]. Other causes of CT occurrence are infections with viral agents like Classical swine fever virus (CSFV), responsible for type A-I [3]. CT of type A-II is prevalent in piglets worldwide, occurs as a sporadic disease affecting single litters, as an outbreak over several weeks affecting a high proportion of farrowing’s or as an ongoing problem frequently affecting gilt litters [4]. Viral agents responsible for CT A-II were sought for decades. In 2015, the novel divergent porcine pestivirus strain “atypical porcine pestivirus (APPV)” was identified in North America and subsequently also detected in Europe [5–7]. Other closely related strains were termed congenital tremor virus (CTV), because they were detected in piglets clinically affected by CT, creating a synonymous name for the same viruses. Serum of affected piglets was used to inoculate pregnant sows to establish the link between APPV and congenital disease. This infection study could reproduce congenital disorders in the offspring [8]. However, Koch’s postulates remain to be proven for APPV. Recently, a first successful cell culture isolation of APPV was reported [5], which might be the key for infection experiments with a defined inoculum.

It is known that pestiviruses may induce various clinical symptoms depending on virus species and strain, as well as on age and immune status of the respective hosts. Beside acute hemorrhagic disease, as documented for CSFV or highly virulent strains of Bovine viral diarrhea virus II (BVDV-II), an infection with most pestiviruses yields mild or subclinical disease in the immune-competent host [9]. Pestiviral infections during gestation may have a detrimental effect on the embryo or fetus, causing stillbirth, neurological defects or malformations [10]. Dysmyelination or hypomyelinogenesis is a characteristic neural lesion in ovine fetuses infected with Border disease virus (BDV) in the late gestation period [11] and such lesions are frequently associated with CT [12]. The clinical signs and histopathological lesions of the so-called “hairy shaker” lambs substantially improve in a few weeks [13, 14], but the exact mechanisms responsible for congenital hypomyelination after in utero infection have not been discovered to date. Historically, CSFV was the only pestivirus known to cause natural infections with clinical significance in swine [15] usually resulting in different clinical symptoms with high morbidity and mortality. CT is a frequent symptom of congenital CSFV infections of piglets. A first novel “atypical pestivirus” (Bungowannah virus) was found in Australian pigs in 2003 but no link to CT was given. Instead, Bungowannah virus caused stillbirth and sudden death of young piglets [16]. The Bungowannah virus is still circulating at the site of initial discovery, but this virus or relatives were never found at other locations [17].

Here we report on the identification and characterization of atypical porcine pestiviruses in seven Austrian farms affected by CT. Clinical symptoms and the course of disease in single litters were followed up over several parities. Histopathological examination showed that clinical symptoms were linked to characteristic hypomyelination in the CNS. APPV could be isolated and propagated on porcine cell lines and the infection was visualized by immunofluorescence. Prevalence of APPV was analyzed by RT-PCR and APPV specific antibodies were determined with a novel NS3 based blocking ELISA to get insights into APPV epidemiology. Monitoring naturally APPV infected and CT affected piglets over a period of 24 weeks gives evidence for a persistent infection. The first documentation of virus shedding via semen in a meanwhile clinically unsuspicious boar, which had been a shaking piglet before, is of special importance with regard to the epidemiology of APPV in the field.

## Materials and methods

### Sample collection

All animal use protocols employed in this study are approved by the institutional ethics and animal welfare committee and the national authority according to §§26ff (Animal Experiments Act from 2012; BMWF-68.205/0188-WF/V/3b/2015). All APPV strains and sequences analyzed in this study originate from these Austrian field cases. In 2015 and 2016, we investigated the pathogens in five Austrian piglet-producing farms, which were experiencing problems with CT and subsequent growth retardation in rearing piglets (Table 1). These cases of CT were brought to our attention by the responsible veterinarians, which reported on several additional cases distributed all over Austria. None of the farms had used vaccines against CSFV according to the Austrian legislation. Veterinarians of the University Clinic for Swine in Vienna conducted the clinical examination and collected samples for diagnostic evaluation. Furthermore, we included a retrospective evaluation of clinical samples from two previous outbreaks, which occurred in 2013 in farms in lower and upper Austria. The clinical examinations in the affected farms only gave a snap shot of the signs and course of disease in some farms. A continuous monitoring of production performance, mortality and pathogen prevalence was not possible in all farms, since the cases occurred in regular commercial breeding farms. To present a typical example, we investigated the outbreak in farm A in detail regarding production losses, clinical symptoms and pathological findings over a 6 month period. Available data about piglet production before and during the occurrence of clinical signs of CT in the other farms is summarized in Table 2.

**Table 1:** **Synopsis of farms participating in the study**

Farm	Year of CT outbreak	Location	# of piglets in pathological examinations	Data generated in the respective farm
A	2015	Upper Austria	10	Exemplary description and prevalence studies
B	2016	Upper Austria		
C	2016	Upper Austria	3	Genomic APPV sequence and long-term monitoring
D	2016	Upper Austria		
E	2016	Upper Austria		
F	2013 and 2016	Lower Austria	2 (in 2013)	Virus isolation
G	2013	Upper Austria	2 (in 2013)

**Table 2:** **Impact of CT occurrence on piglet production, piglet mortality and the splay legs syndrome**

Farm	WP/S/Y before OCT	WP/S/Y during OCT	Mortality of CT-affected piglets	Splay legs observed in CT-affected piglets
A	25.5–26.5	23.57	Up to 30%	Yes
B	24.5	17.2–19.6	i.d.	No
C	25.6	22.8–24.0	i.d.	Yes
D	26.2	26.0	0.6%	No
E	26.5	23.45–23.86	Up to 25%	Yes
F 2013^a^ (2016)	i.d. (24.1)	i.d. (24.0–24.05)	i.d. (<0.6)	i.d. (no)
G	i.d.	i.d.	i.d.	i.d.

WP/S/Y: weaned piglets per sow and year; OCT: occurrence of congenital tremor; CT: congenital tremor; i.d.: indeterminate, due to inexistent or inconsistent records.

^a^ Material was investigated retrospectively and therefor no accompanying data available.

### Farm A

Farm A is a piglet production site located in the northwestern part of Upper Austria. 105 Large-white × Landrace crossbred sows are managed in a 3-week batch-farrowing interval in seven groups consisting of 15 sows each. Gilts are obtained from a gilt producer. Two teaser boars are kept on site for sow stimulation during artificial insemination. Before entering the production cycle, gilts are housed in a separately managed isolation unit for three to nine weeks, which is managed separately. Here, all gilts are vaccinated against parvovirosis and erysipelas (Parvoruvac®, Merial SAS, Lyon, France) and treated alternately with fenbendazole (Panacur 4%, Intervet, Vienna) and ivermectine (Ivomec, Merial SAS) to prevent introduction of sarcoptic mange and round worms. Serum samples of all gilts are screened for PRRSV antibodies before integration into the sow herd. This herd was free of PRRSV before and after occurrence of CT. An average of 26.0 piglets was weaned per sow and year in farm A before CT symptoms occurred.

### Pathology

A complete necropsy was performed on ten piglets of farm A and seven affected piglets from the other farms. Here, we describe the findings from five clinically affected piglets from farm A and five littermates without symptoms. The piglets were euthanized, a gross pathological examination was performed and samples were taken. For histological examination brain, spinal cord, and organ samples of all piglets of farm A and a healthy control animal from a farm without CT problems were fixed in 10% neutral buffered formalin. Formalin fixed brains were cut into coronary sections of 2–3 mm thickness and embedded in paraffin wax. Organ samples and coronary and longitudinal sections of cervical, thoracic and lumbar spinal cord were cut and embedded in paraffin wax, too. Of all embedded organs 1.5 µm thick sections were cut and stained with hematoxylin and eosin (HE). Furthermore, brain and spinal cord samples were stained with a combination of luxol fast blue and HE (LFB-HE) to determine the extent of myelination. Immunohistochemical investigations using a primary antibody (ab109186, dilution 1:1000; Abcam, Cambridge, UK) for determination of olig-2, a marker for oligodendroglial cells, were performed automatically on an autostainer (Lab Vision AS 360, Thermo Fisher Scientific, Waltham, USA). Briefly, 2 μm paraffin-embedded sections of the piglets’ brains and spinal cords were placed on coated slides and dried to enhance tissue adherence. Antigen retrieval was performed on deparaffinized and rehydrated sections by heating in citrate buffer (pH 6). Endogenous peroxidase activity was blocked by incubation in H_2_O_2_. After application of the primary antibody a polymer detection system (UltraVision LP Large Volume Detection System; Thermo Fisher Scientific), consisting of a universal secondary antibody formulation conjugated to an enzyme-labeled polymer was used. The polymer complex was then visualized with an appropriate substrate/chromogen (diaminobenzidine [DAB]; Labvision/Thermo Fisher Scientific). Subsequently, all sections were counterstained with hematoxylin, dehydrated and mounted.

For transmission electron microscopy, samples of cerebellar white matter, cerebellar peduncles, and medulla oblongata of one clinically affected piglet and one piglet without symptoms from farm A were cut in 1 mm^3^ cubes and fixed in 2.5% neutral buffered formalin and 2.5% glutaraldehyde (Merck, Darmstadt, Germany) in 0.1 M phosphate buffer (Sigma-Aldrich, Vienna, Austria), pH 7.2, at 4 °C for 3 h. Afterwards samples were post-fixed in 1% osmium tetroxide (Merck) in the same buffer at 4 °C for 2 h. After dehydration in an alcohol gradient series and propylene oxide (Merck), the tissue samples were embedded in glycid ether 100 (Serva, Heidelberg, Germany). The ultrathin sections were cut on a Leica Ultramicrotome (Leica Ultracut S, Vienna, Austria) and stained with uranyl acetate (Sigma-Aldrich) and lead citrate (Merck). Ultrathin sections were examined with a Zeiss TEM 900 electron microscope (Carl Zeiss, Oberkochen, Germany) operated at 50 kV.

### Detection of APPV genomes and sequence analysis

Total RNA was extracted from field serum samples, semen, saliva or tissue samples using the QIAamp Viral RNA Mini Kit (Qiagen, Hilden, Germany) according to the manufacturer’s instructions. RNA was eluted in 60 µL RNase free distilled water and directly used for RT-PCR or stored at −80 °C for subsequent analysis. RT-PCR was carried out using the OneTaq One-Step RT-PCR Kit (NEB, Ipswich, USA) or the One Step RT-PCR Kit (Qiagen) according to the manufacturer’s instructions. Several primer pairs (available upon request) that were hybridizing with highly conserved regions in different pestivirus species (BVDV, BDV, CSFV, Bungowannah, and APPV) were designed according to published sequences available in NCBI GenBank. Resulting PCR amplicons with suitable length were subjected to gel electrophoresis, purified by the peqGOLD Gel Extraction Kit (Peqlab, Erlangen, Germany), and sequenced by a commercial provider (Eurofins Genomics, Ebersberg, Germany) using the PCR primers. DNA fragments belonging to APPV were sub-cloned in the pGEM-T easy vector (Promega, Madison, USA).

Sequences of Austrian APPV strains were submitted to GenBank with the provisional entries KX778725 (AUT-2015_A), KX778726 (AUT-2016_B), KX778724 (AUT-2016_C), KX778727 (AUT-2016_D), KX778728 (AUT-2016_E), KX778729 (AUT-2013_F), KX778730 (AUT-2016_F), KX778731 (AUT-2013_G). First analyses were carried out using NCBI’s Basic Local Alignment Search Tool for nucleotides (BLASTn). Phylogenetic pairwise comparison and identity calculations were carried out with CLC Main Workbench 7.6 (CLCBIO, Aarhus, Denmark). Alignments and phylogenetic trees were generated with the software CLC Sequence Viewer 7.6 (CLCBIO) with bootstrap values based on 1000 replicates. For construction of the phylogenetic trees, homologous sequences of APPV and other pestivirus species deposited in GenBank were used as indicated.

### Quantitative reverse transcription-PCR (qRT-PCR)

For the quantification of viremia and virus burden, viral RNA was also extracted with QIAamp Viral RNA Kit (Qiagen) according to the manufacturer’s instructions. RNA was eluted in 60 µL distilled water and 1.25 µL was directly used for amplification with the Invitrogen One-Step qRT-PCR Kit (Thermo Fisher Scientific) on an ABI 7500 cycler (Applied Biosystems, Foster City, USA). The APPV specific primers P1 (5′-AGTTCAGAAATCCGGTAGCTG-3′) and P2 (5′-CTACCAGCCTGAGGTCTTC-3′) were used for amplification and the probe P3 (5′-FAM-GTTTCGACACCAAAGCTTGGGACACTCA-TAMRA-3′) was used for detection. A recombinant bacterial plasmid harboring the sequence encoding the NS5B gene of APPV strain AUT 2016 Farm A was purified using the QIAGEN Plasmid Midi Kit (Qiagen) and spectrophotometrically quantified. The copy number of recombinant plasmids was calculated following the formula: N (molecules per µL) = (C (DNA concentration in µg/µL)/K (fragment size in bp)) × 185.5 × 10^13^ (factors derived from DNA weight, volume and the Avogadro constant). In order to obtain a standard curve, a tenfold dilution series of cDNA was included in the qRT-PCR setup. Cycling conditions were 42 °C 15:00, 95 °C 5:00 and 45 cycles of 95 °C 0:05, 60 °C 0:33 (amplification and fluorescence detection step). Genome copies were calculated by 7500 System SDS Software (Applied Biosystems) based on the standard curve. The genome equivalents from 1.25 µL of the purified RNA were projected to copies per 1 mL serum using the multiplication factor 342.9. Sample concentration during RNA preparation (140/60 µL = 2.3) reduced the volume projection factor (1000/1.25 µL = 800) yielding this factor. The copy number per swab was calculated by multiplying with factor 171.4, because the swab was washed out in 500 µL of buffer (500/1.25 µL = 400, concentration factor 2.3).

### Determination of the genomic sequence of an Austrian APPV strain

The genomic sequence of APPV isolate AUT 2016 Farm C (GenBank KX778724) was determined employing primers designed based on an available APPV genome sequence in GenBank (KU194229.1, primer sequences available upon request) [8]. Resulting PCR products were purified, cloned with the help of T-vectors and sequenced as described in sub-section “Detection of APPV genomes and sequence analysis”. RACE-PCRs were not employed to determine the ultimate 5′- and 3′-termini.

### Recombinant antigens

For expression in *E. coli* the coding sequence of the NS3 helicase of APPV AUT 2016 Farm C (AA1513-2006) was amplified by RT-PCR and inserted into a modified pet11a vector (Novagen) with a C-terminal polyhistidine tag (petNS3H-APPV). After expression via a T7 RNA polymerase promoter in *E. coli* strain Rosetta 2™ (Novagen) the protein was purified by ion metal affinity chromatography (IMAC) using Ni^2+^ Sepharose (HisTrap™; GE Healthcare). Amount and purity of APPV-NS3H was accessed by sodium dodecyl sulphate polyacrylamide gel electrophoresis (SDS PAGE), and identity was confirmed by immunoblot analysis with an anti-His antibody. Purified proteins were further purified by size exclusion chromatography (Superdex 200 10/300, GE) and served as antigen source for ELISA studies. The APPV NS3 helicase sequence was sub-cloned in a pcDNA3.1 vector for eukaryotic expression and immunofluorescence tests (pcDNAGFP-NS3H).

### Monoclonal antibodies against pestiviral proteins

As reported earlier, we generated a panel of monoclonal antibodies against the nonstructural proteins of CSFV using heterologous protein expression in the E. coli and standard technics [18]. Over the last years this panel was extended to antibodies against the nonstructural proteins of BVDV [19] and Bungowannah virus (unpublished). In addition, we generated monoclonal antibodies against the structural proteins of CSFV [20] and BVDV [21, 22]. Briefly, BALB/c mice were immunized with recombinant proteins until seroconversion was observed. Spleen cells were prepared and fused with sp2/0-AG14 myeloma cells to generate monoclonal antibody producing hybridomas. Finally, secreted mAbs were evaluated using ELISA, immunoblot and immunofluorescence assays as needed. Each of these fusion experiments yielded up to 100 different reactive monoclonal antibodies. This extensive collection of antibodies against all pestiviral proteins is screened for APPV cross-reactive antibodies using different assays in an on-going project. Until now, we successfully screened different anti-NS3-helicase panels and found two APPV cross-reactive antibodies. Both antibodies were generated by simultaneous immunization with CSFV and BVDV NS3H.

### Serological reagents and ELISA studies

ELISA screening of different hybridoma cell culture supernatants from our library of cross-reactive monoclonal antibodies (mAbs) against pestiviral NS3H was performed as described previously [23]. Briefly, purified recombinant APPV-NS3H was dissolved in ELISA coating buffer (0.1 M sodium carbonate, pH 9.5) and diluted to a final concentration of 10 μg/mL. 96-well ELISA plates (Maxisorb™; Nunc) were coated with 0.5 µg of the APPV-NS3 and blocked with phosphate buffered saline containing 0.01% Tween 20 and 10% fetal calf serum (FCS Gold Plus, Bio&Sell, Feucht, Germany). After incubation with hybridoma cell culture supernatant, specific binding of mAbs was detected with horseradish peroxidase (HRP)-conjugated goat anti-mouse immunoglobulin (Dianova, Hamburg, Germany) and 3,3′,5,5′-tetramethylbenzidine (TMB; Sigma) substrate. The specificity of mAbs was further analyzed in immunoblots and immunofluorescence using the recombinant protein.

The presented indirect ELISA system was modified to analyze the ELISA blocking activity of serum samples from APPV infected pigs. After coating with APPV-NS3 the ELISA wells were blocked either with FCS as a mock control or with the swine sera to be tested. The Cut-off value at a relative signal intensity of 0.5 was determined empirically using defined positive and negative sera. APPV antibody negative sera were obtained from sows and piglets of unsuspicious farms that never experienced problems with CT and were tested negative for APPV by RT-PCR. Defined positive sera originate from sows and piglets from affected farms with high indirect ELISA titers. APPV-NS3H specific antibodies from reactive sera were further characterized in immunofluorescence assays using BHK cells transient transfected with a pcDNA3.1 vector encoding green fluorescent protein fused to the NS3 helicase domain of APPV (pcDNAGFP-NS3H). Reagents suitable for immunofluorescence detection of APPV infected cultured cells were prepared by affinity purification of the porcine APPV NS3H specific antibodies from sera. One milligram of recombinant APPV NS3H was coupled to a NHS-column (HiTrap NHS-activated HP; GE Healthcare, Chalfont St Giles, UK) according to the manufacturer’s recommendations. Ten milliliter of an APPV positive porcine serum was diluted 1:5 in Tris buffer (100 mM, pH: 8.0) and passed over the APPV NS3H-coated matrix. Unbound antibodies were removed and the bound antibody fraction was eluted using a glycine buffer (50 mM, pH: 3.0) and concentrated by ultrafiltration.

### Virus isolation

For virus isolation, 50 µL serum of diseased piglets from different Austrian farms were used to inoculate 5 × 10^6^ SK-6 or PK-15 cells in a six-well format. After 3 days, the infected cells were passaged and a tenth of the cell culture supernatant was further passaged on the same cell line. All cells were grown in Dulbecco’s modified Eagle’s medium (DMEM) supplemented with 10% fetal calf serum (FCS Gold Plus, Bio&Sell) and maintained at 37 °C and 5% CO_2_. Virus infections and passages were analyzed by immunofluorescence and qRT-PCR.

## Results

### Exemplary description of the CT outbreak in farm A

In farm A, the occurrence of CT was first observed in July 2015 in suckling piglets. About 70% of all newborn piglets showed various degrees of trembling or shaking, with 80% of the litters being affected (Table 3). The only symptom visible in most piglets was head shaking, while trembling of the whole body was noticed in severely affected animals (Additional file 1). Stress factors induced or increased shaking symptoms significantly, while no shaking or solely minor tremor was observed during relaxation or sleep. Severely affected newborn piglets were incapable of sucking milk resulting in mortalities of up to 30%. In single piglets a fatal combination of CT and splay legs was observed. During the outbreak of CT between July 2015 and January 2016 productivity dropped from 25.5–26.5 to 23.57 weaned piglets per sow and year. After the outbreak had ceased, productivity levels returned to 25.5–26.5 weaned piglets per sow and year.

**Table 3:** **Course of reproductive performance of sows (mean weaned piglets per sow and year) of farm A and the percentage of CT affected piglets in each farrowing batch**

Farrowing group	Date of parturition	% of piglets affected by CT	Mean # of weaned piglets per sow
2	16.07.2015	70	11.43
3	07.08.2015	80	10.73
4	28.08.2015	20	9.07
5	18.09.2015	80	8.33
6	10.10.2015	30	9.92
7	29.10.2015	25	10.93
1	21.11.2015	30	11.00
2	09.12.2015	0.8	9.13
3	01.01.2016	30	9.71
4	22.01.2016	0	11.20
5	11.02.2016	0	11.64
6	03.02.2016	7	10.64

### Pathology

**Figure 1:**
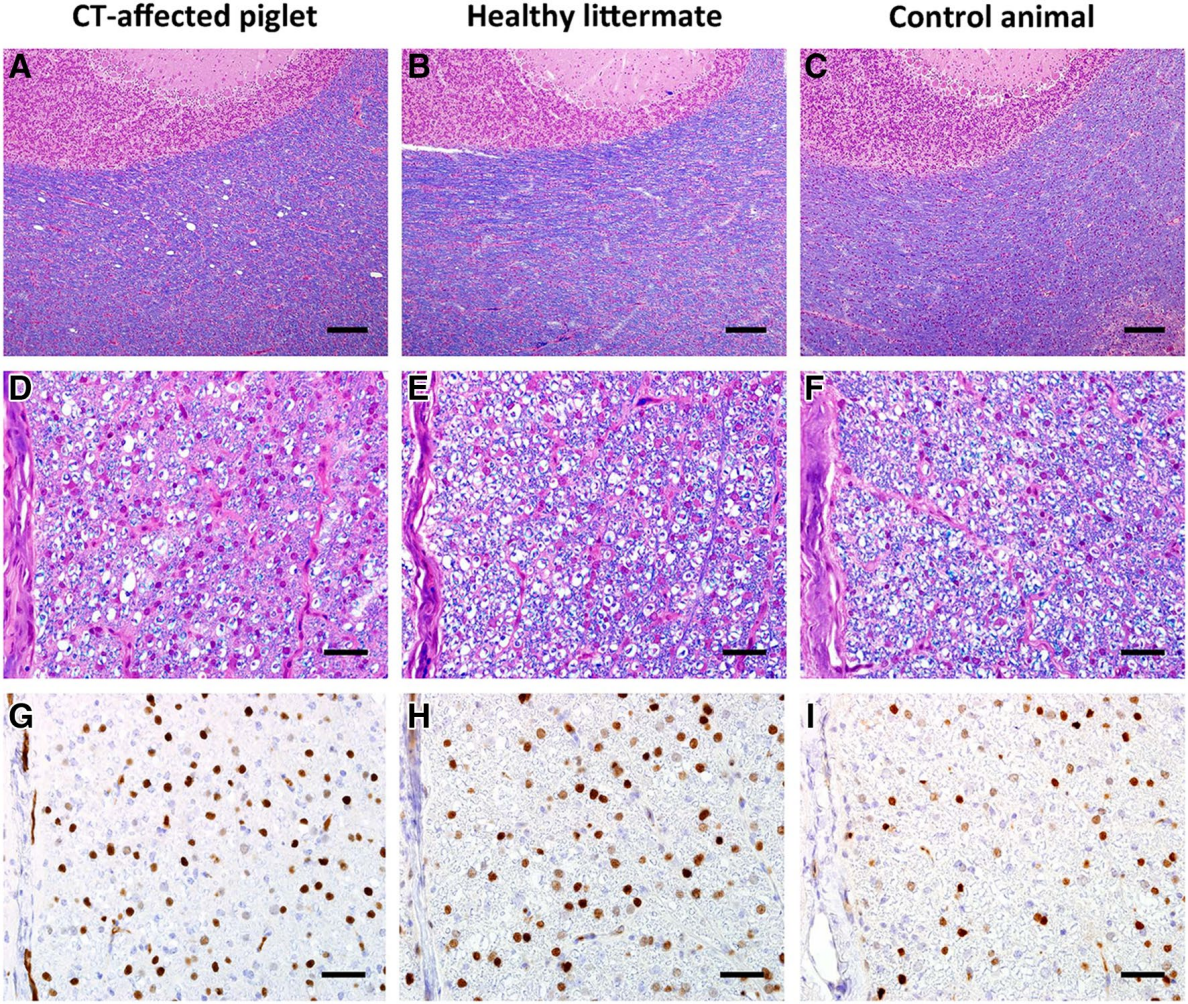
**Histological lesions in a CT-affected animal compared to a healthy littermate and a healthy control.** Vacuoles in cerebellar white matter in affected animal (**A**), normal white matter in littermate (**B**) and control (**C**), LFB-HE, bar = 150 µm. Hypomyelination in white matter of the thoracic spinal cord in affected animal (**D**), normal myelination in littermate (**E**) and control (**F**), LFB-HE, bar = 40 µm. Detection of oligodendrocytes, increased staining intensity in affected animal (**G**), less intense staining in littermate (**H**) and control (**I**), Olig2-IHC, bar = 40 µm.

Ten animals of farm A, five clinically affected and five clinically healthy littermates, were examined and compared to a healthy control animal from a farm without CT problems. On gross examination the piglets showed no severe lesions. Excoriations of the legs, alveolar lung edema and emphysema were present frequently. Four animals out of ten—two affected, two not affected—showed scattered petechiae in the renal cortex. In all animals, brain and spinal cord showed no obvious lesions on gross examination. In the majority of clinically affected animals (*n* = 4/5) few vacuoles were present in the white matter of the cerebellum in LFB-HE-staining, while in the unaffected littermates vacuoles were extremely rare or absent (Figures 1A and B). In the healthy control animal no vacuoles were found (Figure 1C). There were no detectable differences regarding myelination of cerebral and cerebellar white matter in affected and unaffected animals compared to a healthy control (Figures 1A–C). However, hypomyelination was evident in the white matter of the spinal cord of affected animals compared to unaffected littermates and a healthy control (Figures 1D–F). Affected animals showed a clear reduction in the thickness of the myelin sheaths. In one affected animal few dilated myelin sheaths and vacuoles in the white matter of medulla oblongata and spinal cord were found. Unaffected animals did not show any lesions in medulla oblongata or spinal cord. Two affected piglets and one unaffected animal showed mild, partially perivascular, focal gliosis in brain and spinal cord. In the CNS and the kidneys small foci of extravasation were present in affected and unaffected animals from farm A.

Nuclei of oligodendrocytes were labeled in the white matter of affected and unaffected animals as well as the control animal by immunohistochemistry. Neither affected nor unaffected piglets showed reduced numbers of oligodendrocytes compared to the healthy control. In contrast staining intensity of oligodendrocytes was slightly increased in affected animals compared to the healthy control (Figures 1G–I).

For transmission electron microscopic examination two piglets from farm A (one affected, one clinically unaffected) were used. Tissue samples from the cerebellar white matter, cerebellar peduncles and medulla oblongata of both piglets revealed ultrastructural defects such as alterations of myelin and a variety of membrane-bordered vacuoles or spaces in the cytoplasm of glial cells and within axons. In the cerebellar white matter and medulla oblongata samples of the clinically affected piglet, mild hypomyelination accompanied by myelin breakdown and disruption could be found (Additional files 2A and B). There was also multifocal general separation and severe decompaction of myelin lamellae, formation of myelin balloons and degeneration of axons (Additional files 2A–C). Additionally, intramyelinic vacuoles containing membranous debris, which may represent degenerating dendrites or neuronal cell bodies, were evident. In the cerebellar and medulla oblongata samples of the unaffected littermate only mild ultrastructural alterations, such as vacuole formation, hypomyelination and pathological changes in the myelin sheath could be observed.

### Detection of APPV

Using conventional RT-PCR and qRT-PCR targeting the NS5B region we diagnosed the presence of APPV in two retrospective cases (2013) and in six current cases (2015–2016) of CT A-II in Austria. One case occurred 2016 in farm F that had already experienced problems in 2013. More than twenty clinical samples (serum, saliva and CNS material) of healthy pigs from Austrian pig farms, which had never experienced problems with CT, were negatively tested for APPV RNA. APPV was consistently detectable in serum and saliva of CT affected piglets. All sampled organs of two three-week old piglets were positive in conventional RT-PCR, except for the spleen. Serum samples of all sows and boars of farm A were tested negative for the presence of APPV nucleic acid by RT-PCR. In a different farm (farm F), we obtained positive RT-PCR results in the saliva of one sow shortly after farrowing of shaking piglets that were APPV positive. These nucleic acids most likely represent viral contaminations of the sow’s oral cavity obtained from their infected offspring, as the same animal was negatively tested after weaning.

### Characterization of APPV sequences

**Figure 2:**
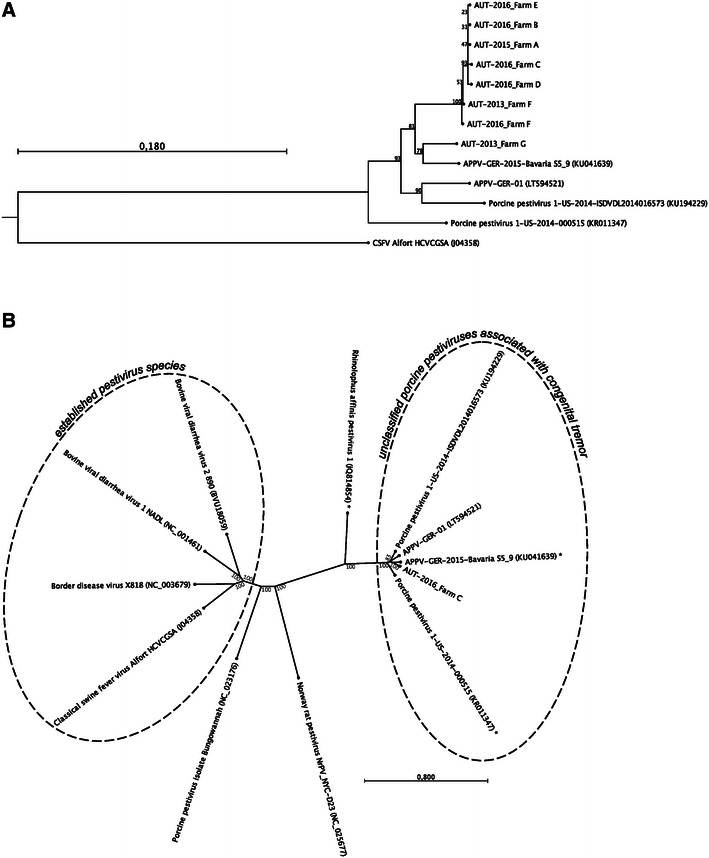
**Phylogeny of Austrian APPV strains.** Phylogenetic trees were constructed using the neighbor joining method with 1000 replicates. **A** Phylogenetic pairwise comparison of NS5B sequence fragments (770 bps) indicates that APPV strains from Austria are closely related to each other and distinct from recently detected APPV strains from the US and Germany. The tree is rooted to CSFV, which serves as an out-group. **B** Simulation of the phylogenetic relationship of available full-length APPV sequences to the four pestiviral species and ungrouped pestivirus isolates (unrooted tree). Full genomes and truncated genome sequences (indicated by asterisk) branch with bootstrap values of >85% each. The GenBank accession numbers of APPV sequences presented here are: KX778725 (AUT-2015_A), KX778726 (AUT-2016_B), KX778724 (AUT-2016_C), KX778727 (AUT-2016_D), KX778728 (AUT-2016_E), KX778729 (AUT-2013_F), KX778730 (AUT-2016_F), KX778731 (AUT-2013_G).

Initial genetic typing of the field strains was done on the basis of a 770 bp fragment of the NS5B genes. All Austrian strains, except one, were closely related to each other (>99% sequence identity, Figure 2A). Only the APPV strain originating from farm G was more distant, clustering with the corresponding NS5B fragment of an APPV strain from Germany (94% sequence identity, Figure 2A). Interestingly, one farm already clinically affected in 2013 (AUT-2013-Farm F) experienced another outbreak in 2016 (AUT-2016-Farm F) with a very closely related strain (one nt exchange). Significant differences to APPV sequences originating from the US (<91% sequence identity, Figure 2A) were observed. A total of 11.535 nucleotides (nt) were determined for APPV strain AUT 2016 Farm C consisting of 360 nt of the 5′NTR, 268 nt of the 3′NTR and 10908 nt of the coding region (ORF). The 5′-NTR misses 10 nt at the 5′-end compared to the APPV from Iowa ISDVDL2014016573 (KU1942299). The open reading frame contains 3635 codons, as for all other available APPV full-length sequences, and yields an amino acid identity of 97.2% (nucleotide identity 93.3%) with the Bavarian strain S5_9 (20), 95.7% (nucleotide identity 89.8%) [5], 94.3% (nucleotide identity 87.2%) with the APPV strain from Kansas, and 95.6% (nucleotide identity 90.0%) with the APPV strain from Iowa. As already reported by others, the distance to the classical pestivirus species (Figure 2B) is considerable with less than 50% nucleotide identity.

### Serological reagents and APPV NS3 blocking ELISA

**Figure 3:**
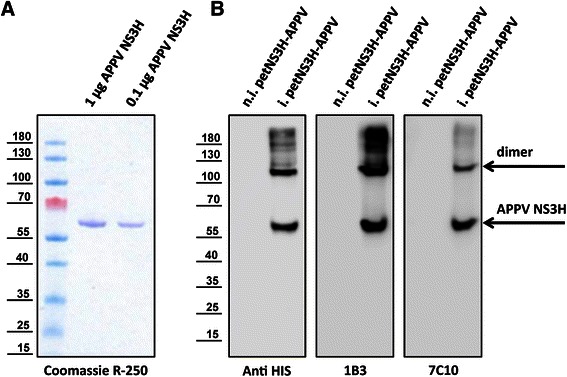
**Purification of recombinant APPV NS3H and reactivity of mAbs. A** Coomassie blue R-250 stain of the recombinant APPV NS3H protein preparation (calculated molecular mass of NS3H 57.9 kDa). **B** Lysates of non-induced (n.i. petNS3H-APPV) and APPV NS3H expressing bacteria (i. petNS3H-APPV) were probed with different antibodies as indicated.

A panel of 120 cross-reactive NS3H specific mAbs, which were established earlier (unpublished data) using recombinant helicase domains of different pestivirus species (CSFV, BVDV-1, BVDV-2, and Bungowannah), was tested for reactivity against the recombinant APPV NS3 helicase (Figure 3A). The NS3H specific antibodies were screened for cross-reactivity using an indirect ELISA. Several antibodies were identified that reacted with the APPV NS3 helicase. The specificity of antibodies WLAPPV3H1 (1B3) and WLAPPV3H2 (7C10) was confirmed in western blot analysis using crude bacterial lysates. Reactivity with specific protein bands at about 60 kDa after induction of NS3 expression is shown in Figure 3B. Unfortunately, neither of these antibodies was suited for the detection of APPV infection by immunofluorescence. Recombinant APPV GFP-NS3H expressed in BHK cells served as positive control but was not recognized (data not shown). We also tried to use APPV GFP–NS3H expressing cells for the screening of porcine sera for the presence of APPV antibodies. This approach failed due to strong unspecific binding of porcine serum antibodies to BHK cells.

**Figure 4:**
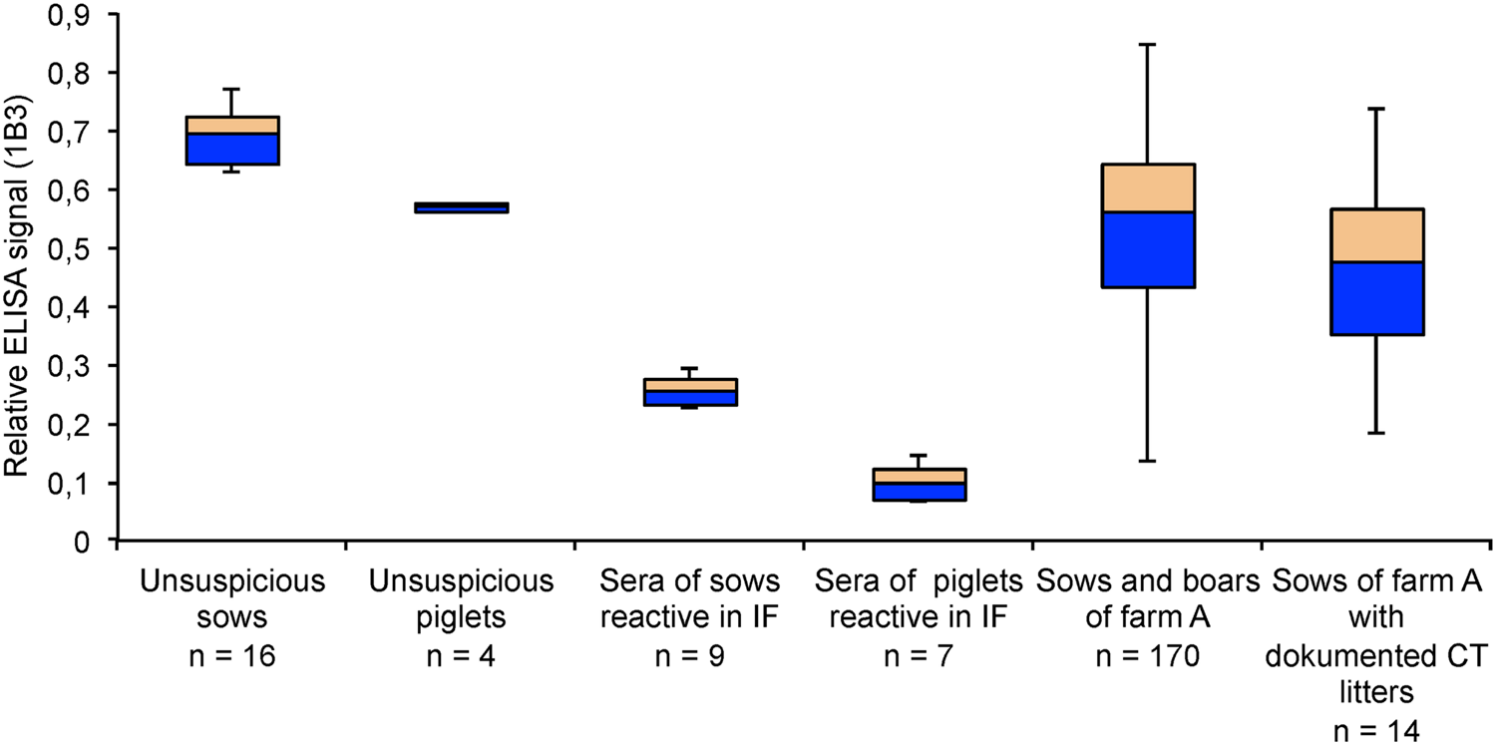
**NS3 blocking ELISA results of farm A.** The relative signal of the mAb 1B3 is depicted after blocking with porcine sera. Blocking with FCS was used as a reference. Sera of unsuspicious sows and piglets from unaffected farms showed relative intensities >50%. Seroconverted sows that had given birth to APPV infected piglets and their CT-affected piglets have a strong serum blocking activity with signal intensities <30%. The specificity of the ELISA reaction was validated by IF staining of cell expressing APPV-NS3H (Additional file 3). The ELISA intensities of all sows and boars of farm A are shown to demonstrate the mixed reactivity within this affected herd. In addition, the ELISA values of 14 sows, which had a litter with well-documented APPV positive CT cases, are presented separately. The sows with APPV associated CT-litters exhibit a stronger reactivity in the blocking ELISA on average. Using a cut-off value of 50% the seroprevalence in farm A reached 35% (60/170).

We applied the recombinant APPV NS3H to test different swine sera in an indirect antibody ELISA setup. Sera of RT-PCR negative sows, which had given birth to clinically affected RT-PCR positive piglets, showed strong ELISA signals, while sera of swine originating from unsuspicious farms demonstrated a weak reactivity. Nevertheless, the specificity and sensitivity of the indirect ELISA was insufficient for further use. To set up a more robust assay, the NS3 specific antibodies 7C10 and 1B3 were employed to establish an APPV NS3H blocking ELISA. Only mAb 1B3 gave satisfactory results with regard to sensitivity and specificity when applied in this type of assay. Using the APPV antibody blocking ELISA we analyzed the prevalence of APPV antibodies in farm A (Figure 4). Sows that had given birth to tremor-affected piglets and had a strong reactivity in the indirect ELISA were used as reactive controls. In addition, we included CT affected piglets with high OD values in indirect ELISAs as reactive controls. All of these sows and piglets were highly positive (OD 0.05–0.3) in the NS3H blocking ELISA. Among the 168 sows and two boars of herd A that were tested, 59 sows and one boar displayed reactivities lower than the threshold (OD 0.5) giving a rate of 35.3% APPV NS3H antibody positive pigs. In addition, we found that sows that had given birth to CT piglets displayed a higher blocking activity than the sows of the affected farm on average. Affinity purified IgGs from blocking ELISA positive animals yielded clear signals in immunofluorescence assays using APPV GFP-NS3H transfected BHK cells as positive control (Additional file 3).

### Isolation and propagation of APPV

**Figure 5:**
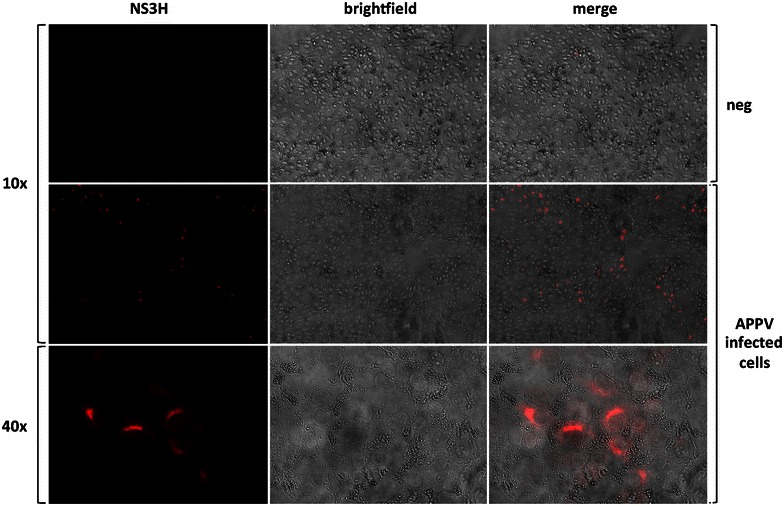
**Detection of APPV infected PK15 cells.** A porcine anti APPV serum purified by NS3 affinity chromatography and a goat-anti swine Cy3 labelled polyclonal antibody was used for fluorescence staining. Cy3 fluorescence, brightfield and merge images are shown for uninfected and infected cells at 10× magnification. To resolve the perinuclear staining pattern, a cluster of positive cells is also shown at 40× magnification.

The availability of APPV specific antibodies as well as qRT-PCR assay allowed us to screen for APPV infection of cultured cells. After initial attempts to isolate APPV had failed and thus confirmed reports of others [6–8], we could show the presence of APPV antigen in SK-6 and PK-15 cells after 5 passages by immunofluorescence (Figure 5) and qRT-PCR. Key to this experiment was the inoculation of cells with an APPV positive serum sample (APPV AUT 646/16) that was obtained from a CT piglet before suckling and hence devoid of colostral antibodies. In contrast to high nucleic acid titers measured in the samples of APPV positive piglets (>10^6^ GE/mL or swab) and in the cell culture supernatant (>10^9^ GE/mL), the infectivity of these samples was minute (10^1^–10^2^ ffu/mL). Virus spread of the APPV isolate in cell culture was very inefficient as indicated by the small size of antigen positive foci (Figure 5).

### Long-term monitoring of APPV infected piglets in farm F

**Figure 6:**
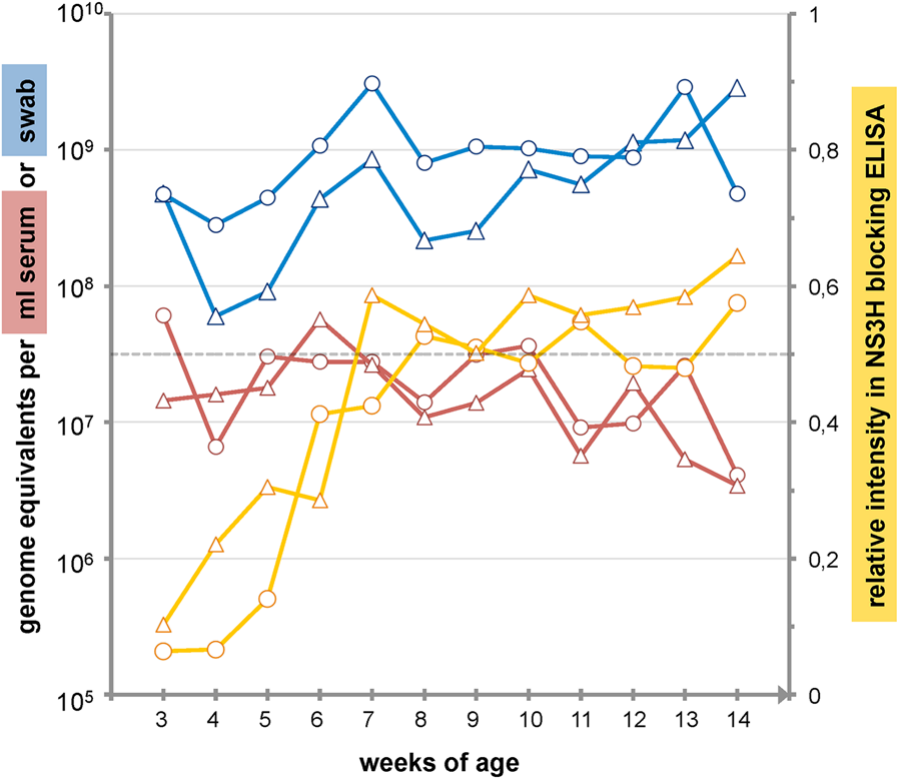
**Course of APPV infection in two CT affected piglets.** qRT-PCR values of serum samples (red) and oral swabs (blue) are depicted together with the relative signal intensities of the NS3 blocking ELISA (yellow). The dotted grey line represents the empirical cut-off value of 0.5. Note the constant presence of APPV genomes in both animals in serum and saliva over the period of 12 weeks, whereas APPV specific antibodies could no longer be detected at the age of 8 weeks indicating maternal origin.

Five RT-PCR positive shaking piglets from one affected litter at farm F were chosen for detailed analyses of disease. Three of the piglets were sacrificed at different time points for storage of organ material. For integration in the herd, the health status of one female and one male piglet was assessed over 6 months and consecutive diagnostic tests were conducted to determine the risk of virus shedding and transmission. The piglets showed a mild tremor most obvious at the ear tips and flank as shown in the Additional file 1. CT symptoms improved within several weeks and disappeared completely until 14 weeks of age. The course of viremia, virus shedding via saliva and the APPV NS3H specific antibody titers are presented for both piglets from week 3 to 14 weeks of age (Figure 6). Continuously positive qRT-PCR results in serum and saliva samples demonstrate constant viremia and virus shedding in both animals over the period of 12 weeks. APPV NS3H specific antibodies were present after birth, but vanished in both animals at the age of eight weeks. One of these piglets became a clinically unsuspicious boar that reached sexual maturity. At 6 months of age, high levels of APPV genomes were present in saliva (2.9 × 10^8^ GE/swab) and semen (2.1 × 10^9^ GE/mL), while levels in serum remained lower in the boar (2.0 × 10^7^ GE/mL). The serum APPV genome load of the 6-month-old sow was below the detection limit of our qPCR assay (LOD: 3.0 × 10^6^ GE/mL) in two consecutive tests, while high genome loads could still be observed in saliva (1.3 × 10^9^ GE/swab).

## Discussion

Outbreaks of CT in several piglet producing farms in Austria between 2013 and 2016 were puzzling as comprehensive analyses of non-infectious noxae (mycotoxins) and known porcine pathogens (Herpes-, Entero-, and Sapoviruses) gave negative or inconclusive results. The discrete histologic lesions present in the spinal cord of infected animals were consistent with hypomyelination. These alterations were quite similar to lesions reported in piglets infected with CSFV [3] and a calf infected with BVDV [24]. The mild vacuolization of the cerebellar white matter of affected animals is in accordance with lesions reported in piglets with CT due to suspected BVDV-related pestivirus infection [25]. Immunohistochemically there was no detectable loss of oligodendrocytes in the spinal cord of affected animals. There was rather an increased expression of Olig 2 present in affected piglets. Furthermore, ultrastructural examination of cerebellar white matter showed not only hypomyelination but myelin disruption and breakdown in affected animals. This is in contrast to ultrastructural lesions reported in piglets with classical swine fever [3] and a calf infected with BVDV [24]. We therefore conclude that in utero infection with APPV has a deleterious effect on fetal oligodendrocytes, which are present in sufficient numbers. This effect may have an impact on myelin development and function or lead to degeneration of the present myelin. Further research in this regard is crucial to better understand the pathogenesis of the disease.

We analyzed diagnostic samples of CT-affected piglets employing RT-PCRs using primers deduced from the NS5B region of a published novel atypical pestivirus sequence shortly after its availability by the end of 2015 [7]. In all tested cases we amplified APPV specific RT-PCR products that upon sequencing matched well (about 90%) to the published sequence. Apart from one sequence obtained from a CT case from 2013 (farm G), the Austrian isolates show a very high degree of sequence conservation within the analyzed NS5B fragment (>99%) and form a distinct cluster compared to isolates from Germany and the US (Figure 4A). One farm (F) experienced an outbreak in 2013 and 2016. As the analyzed NS5B fragments only contain 1 nt exchange but differ from the isolates of farm A-E in three conserved residues, viral persistence in this herd is likely. Due to APPV detection on two continents, it seems likely that APPV has a worldwide distribution, especially if one takes into account that the “non-CSFV” clinical picture of CT A-II has been known for decades. APPV most likely has been present in domestic pigs since decades but remained elusive, as diagnostics targeting classical pestiviruses do not cross-react.

While detection of APPV genomes by PCR is straightforward and different RT-PCR protocols have already been developed targeting conserved sites within NS3 or NS5B, there is only one report about the establishment of an E^rns^ based serological assay. Hause et al. determined antibodies against recombinant bacterially expressed APPV E^rns^ in 94% of randomly tested animals, while sera from a specific pathogen free farm yielded no positive reactions [7]. We established a serological assay on the basis of NS3 in analogy to NS3 blocking ELISAs routinely used in CSFV and BVDV serodiagnostic. First indirect ELISA tests with recombinant APPV NS3H coated to plates were sufficient to discern clearly positive (some sows that farrowed CT piglets) from clearly negative sera (non-infected controls from APPV unsuspicious farms) but a more detailed analysis was not possible. NS3 contains at least two indispensable enzymatic functions, protease and helicase and functional protein can easily be produced in *E. coli*. In previous projects we characterized a panel of 120 cross-specific mAbs against NS3 molecules of various pestiviruses including BVDV-II and Bungowannah virus. We screened these mAbs against the bacterially expressed APPV NS3 helicase domain (60 kDa) using ELISA. One mAb proofed suitable for the application in a highly specific blocking ELISA. In our survey of farm A, which had experienced several farrowings with clinical overt CT in APPV positive piglets, but revealed no APPV RT-PCR positive adults, the reactivity rate was 60/170 (35.3%). These results were quite contrasting the 94% seroprevalence reported earlier in samples initially taken for a PRRSV survey employing an E^rns^ based ELISA. E^rns^ is a highly glycosylated protein with several intramolecular disulfide bonds. Therefore, it is possible that antigen produced in bacteria may not be optimal for diagnostic purposes. Beside the test system, the different APPV strains might vary in their immunological properties explaining differences in seroprevalence between Austria and the US. Future studies will have to compare the reactivities of both ELISA concepts. APPV antigen detection in infected cells or tissues was difficult and required the preparation of NS3 monospecific antibodies extracted from APPV positive pig sera. This procedure was necessary because the NS3 specific mAbs did not react in immunofluorescence or immunohistochemistry applications. The monospecific porcine NS3 antibodies were reactive with APPV infected cultured cells but were not applicable in porcine tissues due to high background caused by the intrinsic presence of porcine IgG. In our hands in situ hybridization also failed to detect APPV genomes in tissues. Likely our method is not sensitive enough and an optimized FISH protocol (as described by Postel et al. [16]) needs to be applied.

As reported earlier [6, 8, 26], clinical signs of CT can be dramatic in piglets, but cease over time. The Austrian cases described in this study were associated with moderately increased piglet mortality. Losses of 2.5 piglets per sow and year in farm A mainly occurred as severe tremor interfered with the piglets’ ability to suckle milk. The farmers also reported on some growers with persistent tremor that decreased the productivity. Not surprising for a newly discovered agent, routes of infection, epidemiology of APPV and pathogenesis of CT remain unclear. The evidence for APPV as a causative agent behind CT is robust but Koch’s postulates have not been fully fulfilled, yet [8]. Recent infection experiments indicated a trans-placental infection of piglets. Controlled animal experiments are essential to evaluate the clinic signs of intrauterine APPV infection, the impact of APPV on piglet production and the economic importance of this virus. Nevertheless, virus isolation is key to address these questions. As we have shown that APPV can be propagated and persists in porcine cells, animal trials to proof Koch’s postulates can now be conducted. Also, cultured isolates are a basic requirement to address questions related to the molecular biology of APPV, such as its low infectivity in vitro or the role of the N-terminal E2 truncation [7].

Up to date we were not able to identify the source of virus introduction into the affected Austrian farms. We could show that persistently infected (PI) animals exist, reach sexual maturity and shed high virus quantities with their saliva and semen in the absence of any clinical signs or antibody response. It is not clear from our study, whether the persistence of APPV is a common phenomenon in CT affected animals. By analogy to other pestiviruses we assume that immunotolerance and virus persistence solely occurs after In utero infection within a defined gestation period yielding a few percent of PI animals. These animals are a reservoir for APPV and a threat for APPV naïve herds. Also, as we showed high viral loads in semen of an adult boar formerly affected by CT, there is a potential for sexual transmission or transmission via artificial insemination. This aspect urgently needs clarification, as it could severely affect the pig industry. Usually a farm experiences CT affected farrowings for a few weeks to a few months, depending on the production scheme, including some sows rearing healthy litters before the clinical signs completely subside. This is indicative of a transient infection process that affects only a few animals clinically in the suitable gestation period, while the others are subclinically infected and mount an antibody response. The serological data of our study at farm A using the NS3H based blocking ELISA show that the farrowing of CT affected piglets is not necessarily inducing detectable antibody levels as indicated by the moderate seroprevalence of 35.3%. We can only speculate on the immunogenicity of our target epitope and it is so far unclear whether the antibody response is stringent. The blocking antibody response could also be transient and vanish within a short period of time. A high prevalence of atypical porcine pestiviruses was found in German pig herds with no obvious link to CT [5]. However, within an immune herd APPV might be present in persistently infected animals without inducing clinical signs. This is in line with the hypothesis that CT in piglets only occurs when naïve sows are infected in a certain gestation period [8].

The discovery of APPV demonstrates that the diversity among the genus pestivirus was underestimated and that it is likely that more “atypical” pestiviruses circulate in domestic and feral populations of pigs and ruminants. Novel pestivirus sequences were also reported for rats (Norway rat pestivirus) in North America and bats (*Rhinolophus affinis* pestivirus) in China [27, 28], which are distantly related to both classical and atypical porcine pestiviruses. With regard to nomenclature the proposed terms “atypical porcine pestivirus, APPV” [7] or “congenital tremor virus, CTV” [8] are misleading as “atypical” is a more general term already used for other pestiviruses (e.g. BVDV 3). The same is true for CTV, as CT has earlier been linked to certain strains of CSFV or BDV in lambs. As it can be expected that the genus pestivirus will expand in the near future, a more rational terminology would be straightforward. As in herpesviruses (human herpesvirus 1–8) the host species could be combined with a number. According to this strategy CSFV could be termed as Porcine pestivirus 1, Bungowannah virus as Porcine pestivirus 2 and Atypical porcine pestivirus as Porcine pestivirus 3 based on the order of first description. Strict rules for species affiliation regarding sequence identities would further substantiate the concept. Besides terminology, this would also simplify the separation of notifiable and non-notifiable livestock diseases caused by pestiviruses. The biology of APPV, considering its pestivirus-specific traits, such as persistence or in utero infection, as well as its unique properties, such as high genome loads in saliva versus lower to non-detectable levels in serum, points out that there is much more variety than expected within the genus pestivirus.

## Additional files

**Additional file 1.** **Movie of CT-affected piglets of farm A.** Note the tremor most obvious at the ear tips and flank.

**Additional file 2.** **Electron micrographs of the central nervous system of an affected piglet show severe lesions.** (A) Separation and decompaction of myelin sheaths (white asterisk) as well as intraaxonal vacuole formation (x) in the cerebellar white matter, bar = 2.5 µm. (B) Axonal degeneration (black arrow), vacuole formation (x) and myelin balloons (black asterisk) in the medulla oblongata, bar = 2.5 µm. (C) Defects of the myelin lamellae characterized by disruption of lamellae (black arrowhead) and formation of myelin balloons (black asterisk) in the medulla oblongata, bar = 250 nm.

**Additional file 3.** **Validation of NS3H blocking ELISA results.** BHK cells expressing GFP-NS3H were incubated with NS3H affinity purified porcine sera and stained by Cy3 conjugated goat anti swine serum. Blocking ELISA positive sows (#1–3) and a negative sow #4 are shown. A brightfield image of the BHK cells (left panel), the GFP (middle panel) and the Cy3 fluorescence (right panel) are presented. A murine anti GFP antibody is applied as a positive control.

## References

[1] Blakemore WF, Harding JD, Done JT (1974). Ultrastructural observations on the spinal cord of a Landrace pig with congenital tremor type AIII. Res Vet Sci.

[2] Blakemore WF, Harding JD (1974). Ultrastructural observations on the spinal cords of piglets affected with congenital tremor type AIV. Res Vet Sci.

[3] Bradley R, Done JT, Hebert CN, Overby E, Askaa J, Basse A, Bloch B (1983). Congenital tremor type AI: light and electron microscopical observations on the spinal cords of affected piglets. J Comp Pathol.

[4] Done JT, Woolley J, Upcott DH, Hebert CN (1986). Porcine congenital tremor type AII: spinal cord morphometry. Br Vet J.

[5] Beer M, Wernike K, Drager C, Hoper D, Pohlmann A, Bergermann C, Schroder C, Klinkhammer S, Blome S, Hoffmann B (2016). High prevalence of highly variable atypical porcine pestiviruses found in Germany. Transboun Emerg Dis.

[6] Postel A, Hansmann F, Baechlein C, Fischer N, Alawi M, Grundhoff A, Derking S, Tenhundfeld J, Pfankuche VM, Herder V, Baumgartner W, Wendt M, Becher P (2016). Presence of atypical porcine pestivirus (APPV) genomes in newborn piglets correlates with congenital tremor. Sci Rep.

[7] Hause BM, Collin EA, Peddireddi L, Yuan F, Chen Z, Hesse RA, Gauger PC, Clement T, Fang Y, Anderson G (2015). Discovery of a novel putative atypical porcine pestivirus in pigs in the USA. J Gen Virol.

[8] Arruda BL, Arruda PH, Magstadt DR, Schwartz KJ, Dohlman T, Schleining JA, Patterson AR, Visek CA, Victoria JG (2016). Identification of a divergent lineage porcine pestivirus in nursing piglets with congenital tremors and reproduction of disease following experimental inoculation. PLoS One.

[9] Bielefeldt Ohmann H (1988). BVD virus antigens in tissues of persistently viraemic, clinically normal cattle: implications for the pathogenesis of clinically fatal disease. Acta Vet Scand.

[10] Barlow RM (1980). Morphogenesis of hydranencephaly and other intracranial malformations in progeny of pregnant ewes infected with pestiviruses. J Comp Pathol.

[11] Anderson CA, Higgins RJ, Smith ME, Osburn BI (1987). Border disease. Virus-induced decrease in thyroid hormone levels with associated hypomyelination. Lab Invest.

[12] Clarke GL, Osburn BI (1978). Transmissible congenital demyelinating encephalopathy of lambs. Vet Pathol.

[13] Anderson CA, Higgins RJ, Waldvogel AS, Osburn BI, Barlow RM, Patterson DS (1982). Tropism of border disease virus for oligodendrocytes in ovine fetal brain cultures. Border disesase of sheep; a virus-induced teratogenic disorder.

[14] Anderson CA, Sawyer M, Higgins RJ, East N, Osburn BI (1987). Experimentally induced ovine border disease: extensive hypomyelination with minimal viral antigen in neonatal spinal cord. Am J Vet Res.

[15] Tao J, Liao J, Wang Y, Zhang X, Wang J, Zhu G (2013). Bovine viral diarrhea virus (BVDV) infections in pigs. Vet Microbiol.

[16] Kirkland PD, Frost MJ, Finlaison DS, King KR, Ridpath JF, Gu X (2007). Identification of a novel virus in pigs–Bungowannah virus: a possible new species of pestivirus. Virus Res.

[17] Kirkland PD, Read AJ, Frost MJ, Finlaison DS (2015). Bungowannah virus–a probable new species of pestivirus–what have we found in the last 10 years?. Anim Health Res Rev.

[18] Lamp B, Riedel C, Roman-Sosa G, Heimann M, Jacobi S, Becher P, Thiel HJ, Rumenapf T (2011). Biosynthesis of classical swine fever virus nonstructural proteins. J Virol.

[19] Isken O, Langerwisch U, Schonherr R, Lamp B, Schroder K, Duden R, Rumenapf TH, Tautz N (2014). Functional characterization of bovine viral diarrhea virus nonstructural protein 5A by reverse genetic analysis and live cell imaging. J Virol.

[20] Riedel C, Lamp B, Heimann M, Konig M, Blome S, Moennig V, Schuttler C, Thiel HJ, Rumenapf T (2012). The core protein of classical swine fever virus is dispensable for virus propagation in vitro. PLoS Pathog.

[21] Gilmartin AA, Lamp B, Rumenapf T, Persson MA, Rey FA, Krey T (2012). High-level secretion of recombinant monomeric murine and human single-chain Fv antibodies from Drosophila S2 cells. Protein Eng Des Sel.

[22] Callens N, Brugger B, Bonnafous P, Drobecq H, Gerl MJ, Krey T, Roman-Sosa G, Rumenapf T, Lambert O, Dubuisson J, Rouille Y (2016). Morphology and molecular composition of purified bovine viral diarrhea virus envelope. PLoS Pathog.

[23] Lamp B, Riedel C, Wentz E, Tortorici MA, Rumenapf T (2013). Autocatalytic cleavage within classical swine fever virus NS3 leads to a functional separation of protease and helicase. J Virol.

[24] Porter BF, Ridpath JF, Calise DV, Payne HR, Janke JJ, Baxter DG, Edwards JF (2010). Hypomyelination associated with bovine viral diarrhea virus type 2 infection in a longhorn calf. Vet Pathol.

[25] Segalés J, Granberg F, Liu L, Cabezón O, Rosell R, Belák S (2012). Is congenital tremor type AII of pigs associated to bovine viral diarrhoea virus (BVDV) and/or a BVDV-related pestivirus?. IPVS Congress VO-.

[26] Vandekerckhove P, Maenhout D, Curvers P, Hoorens J, Ducatelle R (1989). Type A2 congenital tremor in piglets. Zentralbl Veterinarmed A.

[27] Wu Z, Ren X, Yang L, Hu Y, Yang J, He G, Zhang J, Dong J, Sun L, Du J, Liu L, Xue Y, Wang J, Yang F, Zhang S, Jin Q (2012). Virome analysis for identification of novel mammalian viruses in bat species from Chinese provinces. J Virol.

[28] Firth C, Bhat M, Firth MA, Williams SH, Frye MJ, Simmonds P, Conte JM, Ng J, Garcia J, Bhuva NP, Lee B, Che X, Quan PL, Lipkin WI (2014). Detection of zoonotic pathogens and characterization of novel viruses carried by commensal Rattus norvegicus in New York City. MBio.

