# Supplementary figures and images for: Congenital infection with atypical porcine pestivirus (APPV) is associated with disease and viral persistence
Source: Vet Res. 2017 Jan 6;48:1. doi: 10.1186/s13567-016-0406-1 (PMC5217315; doi:10.1186/s13567-016-0406-1)

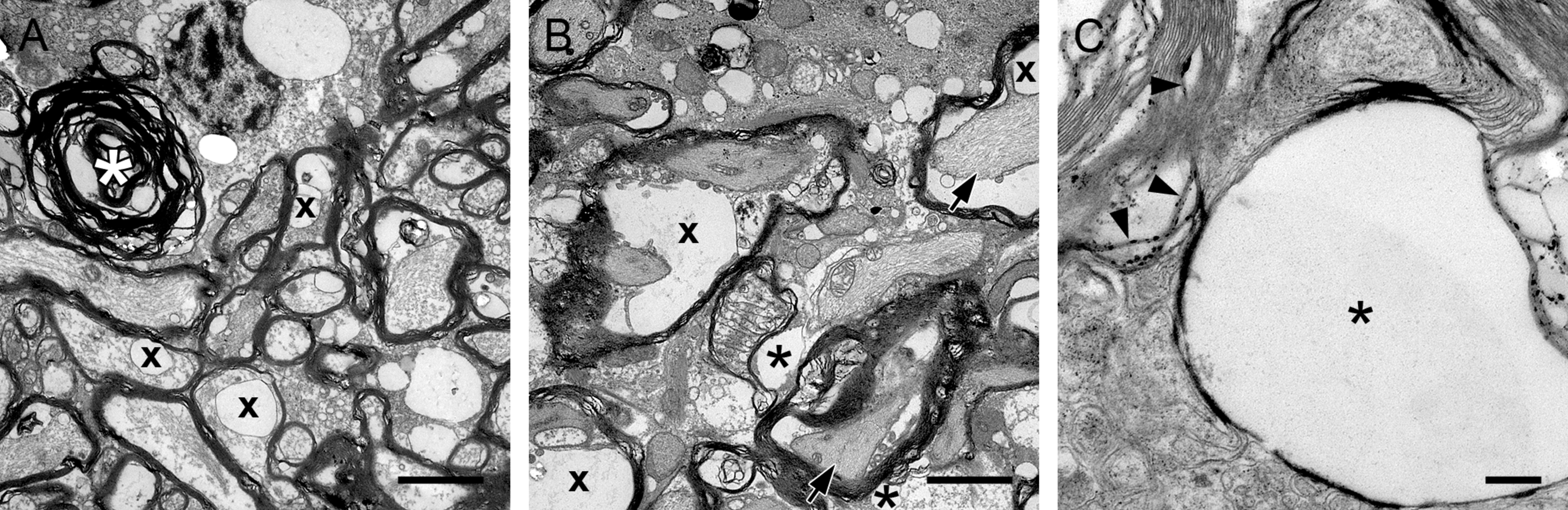

Supplement: Supplementary file 2 — Additional file 2. Electron micrographs of the central nervous system of an affected piglet show severe lesions. (A) Separation and decompaction of myelin sheaths (white asterisk) as well as intraaxonal vacuole formation (x) in the cerebellar white matter, bar = 2.5 µm. (B) Axonal degeneration (black arrow), vacuole formation (x) and myelin balloons (black asterisk) in the medulla oblongata, bar = 2.5 µm. (C) Defects of the myelin lamellae characterized by disruption of lamellae (black arrowhead) and formation of myelin balloons (black asterisk) in the medulla oblongata, bar = 250 nm. [file 13567_2016_406_MOESM2_ESM.tif]

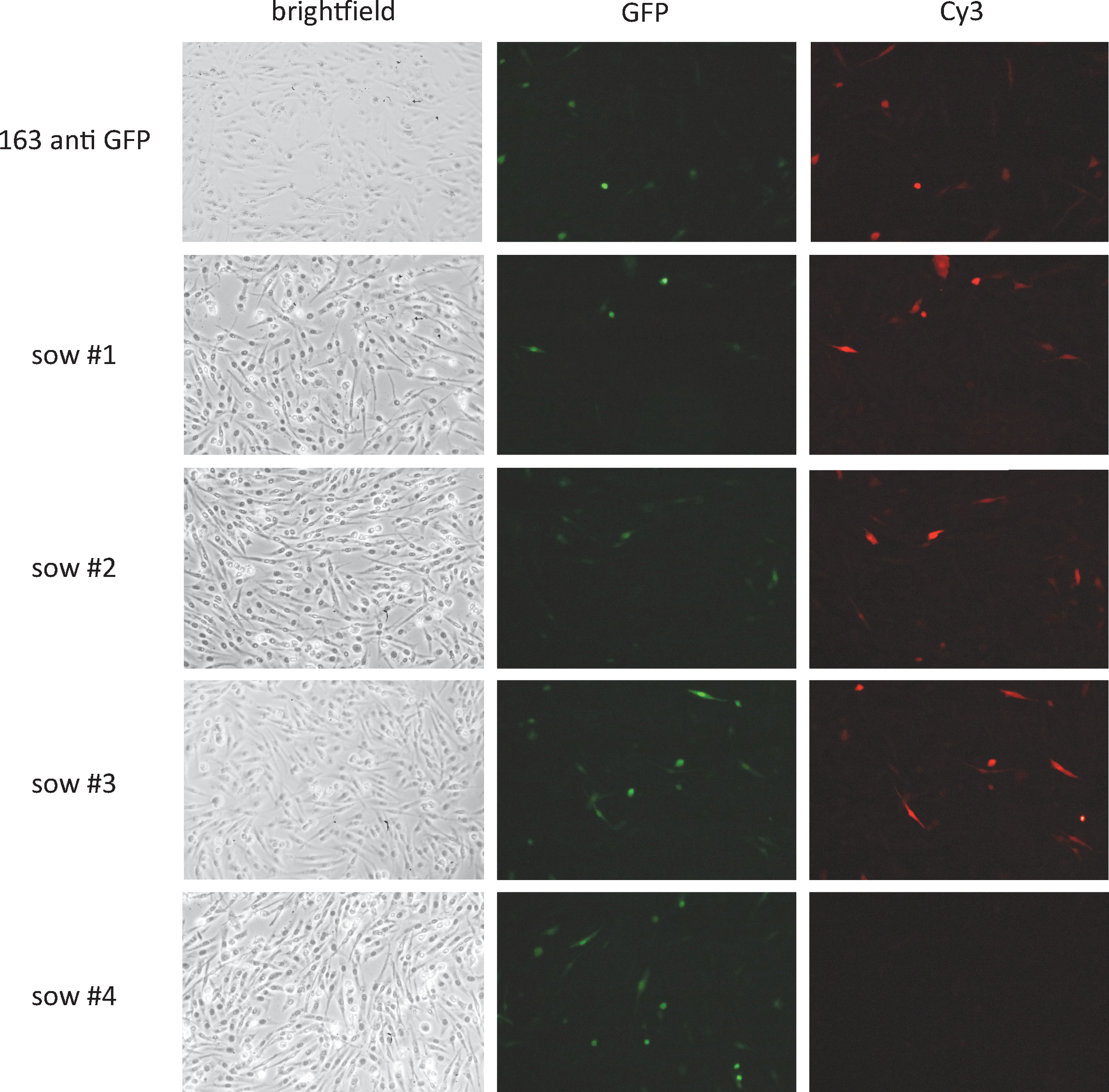

Supplement: Supplementary file 3 — Additional file 3. Validation of NS3H blocking ELISA results. BHK cells expressing GFP-NS3H were incubated with NS3H affinity purified porcine sera and stained by Cy3 conjugated goat anti swine serum. Blocking ELISA positive sows (#1–3) and a negative sow #4 are shown. A brightfield image of the BHK cells (left panel), the GFP (middle panel) and the Cy3 fluorescence (right panel) are presented. A murine anti GFP antibody is applied as a positive control. [file 13567_2016_406_MOESM3_ESM.tif]
